# Strengthening the links between mapping, planning and global engagement for disease elimination: lessons learnt from trachoma

**DOI:** 10.1136/bjophthalmol-2018-312476

**Published:** 2018-06-15

**Authors:** Paul Courtright, Lisa A Rotondo, Chad MacArthur, Iain Jones, Angela Weaver, Biruck Kebede Negash, Nicholas Olobio, Kamal Binnawi, Simon Bush, Mariamo Abdala, Danny Haddad, Astrid Bonfield, Paul Emerson, Virginia Sarah, Anthony W Solomon

**Affiliations:** 1 Kilimanjaro Centre for Community Ophthalmology, Division of Ophthalmology, University of Cape Town, Cape Town, South Africa; 2 RTI International, Washington, DC, USA; 3 Department for International Development, London, UK; 4 United States Agency for International Development, Washington, DC, USA; 5 Federal Ministry of Health, Addis Ababa, Ethiopia; 6 Federal Ministry of Health, Abuja, Nigeria; 7 Ministry of Health and Alneelain University, Khartoum, Sudan; 8 Sightsavers, Accra, Ghana; 9 Nacional Eye Care Program, Ministério da Saude de Mozambique, Maputo, Mozambique; 10 Orbis International, New York, New York, USA; 11 The Queen Elizabeth Diamond Jubilee Trust, London, UK; 12 International Trachoma Initiative, Atlanta, Georgia, USA; 13 International Coalition for Trachoma Control and The Fred Hollows Foundation, London, UK; 14 Department of Control of Neglected Tropical Diseases, World Health Organization, Geneva, Switzerland

**Keywords:** Public health, Epidemiology

## Abstract

**Background:**

Trachoma is the leading infectious cause of blindness. Until recently, reliable data on the global extent of the disease, detailed plans for elimination, and government, donor and partner engagement were all inadequate.

**Methods:**

The trachoma community undertook a systematic, three-pronged strategy to map trachoma district by district, develop national-level trachoma elimination plans, and create a framework for governments, donors and partners to convene and coordinate in support of trachoma elimination.

**Result:**

There has been a frame-shift in internal and external perceptions of the global trachoma programme, from being an effort working towards disease control in focussed geographical areas, to one in the process of achieving worldwide disease elimination. Multiple factors contributed to the successful implementation of mapping, planning, and cross-sectional engagement of governments, partners and donors.

**Conclusions:**

Elimination of trachoma is possible if the right combination of factors is in place. Planning for success is a critical first step. Some remaining challenges must still be addressed if the elimination targets are to be successfully attained.

## Background

Trachoma, caused by *Chlamydia trachomatis*, has been noted throughout history as a significant cause of blindness, yet there is an expectation that by the year 2020 it will have been eliminated as a public health problem from most countries. Trachoma affects rural communities that have inadequate access to water, sanitation and healthcare. In 1998, the World Health Assembly adopted the goal of Global Elimination of Trachoma as a cause of blindness^1^; the year 2020 was set as the target date by a WHO Alliance set up to support the elimination agenda.^2^ These events were accompanied by the global health community adopting the SAFE strategy (Surgery to correct trichiasis, Antibiotics to clear infection, and Facial cleanliness and Environmental improvement to reduce transmission), which is the strategy recommended by WHO.^3^ While some progress was made over the subsequent 12 years,^4^ it was clear that efforts were insufficient to bring about elimination. Barriers to achieving the goal included a lack of reliable data on the magnitude of trachoma and its distribution, insufficient political and financial support, and imperfectly formed elimination plans.

In 2018, the trachoma landscape looks vastly different. Prospects for achieving elimination are more promising. Global mapping of trachoma is almost complete, most trachoma endemic countries have clear and practical plans for implementation and elimination, and governments, donors and partners have significantly increased their support for elimination. Understanding the creation and evolution of the strong links between baseline mapping and planning and government/donor/partner engagement, which occurred between 2012 and now—and how these links set countries on the path to elimination^i^—is the subject of this manuscript.

## Trachoma elimination

Trachoma is a neglected tropical disease (NTD). The causative organism is passed from person to person by flies, fomites and fingers, particularly among preschool-aged children.^5^ Infection is associated with the development of signs of ‘active’ (inflammatory) trachoma in the conjunctivae. After multiple episodes of active trachoma, some people develop trachomatous conjunctival scarring.^6 7^ Conjunctival scarring may deform the eyelid, leading to deviation of the eyelashes so that they abrade the globe, a condition referred to as trachomatous trichiasis (TT).^8^ Corneal opacity, caused by TT, impairs vision. Elimination of trachoma requires interventions against both active trachoma and TT.^9^

In trachoma, prevalence data are used to decide if and how to intervene, when interventions can cease, and when elimination has been achieved.^10 11^ The WHO guidelines for intervention^10^ and validation of elimination^11^ (table 1) have been foundational for elimination programme planning.

**Table 1:** WHO criteria for intervention against and elimination of trachoma as a public health problem

Interventions	Population group surveyed	Sign measured	Decision
**Criteria for initiation of trachoma elimination programmes (district level): baseline survey**			
AFE	Children aged 1–9 years.	TF	<5%=no intervention (active trachoma not a public health problem). 5%–9.9%=1 year of AFE, then impact survey. 10%–29.9%=3 years of AFE, then impact survey. ≥30%=5 years of AFE, then impact survey.
S	Adults aged 15 years and above.	TT	<0.2%=no public health-level intervention (TT not a public health problem). ≥0.2%=community-based TT management programme.
**Criteria for cessation of interventions (district level): impact survey**			
AFE	Children aged 1–9 years.	TF	<5%=discontinue A, maintain F&E. 5%–9.9%=1 year of AFE, then impact survey. 10%–29.9%=3 years of AFE, then impact survey. ≥30%=5 years of AFE, then impact survey.
S	Adults aged 15 years and above.	TT	<0.2%=discontinue community-based TT management; strengthen facility-based management. ≥0.2%=continue community-based TT management programme.
**Criteria for elimination (district level): surveillance survey**			
AFE	<5% TF in children aged 1–9 years.		
S	<0.2% of unmanaged TT in adults aged 15 years and above.*		

*WHO.^26^

AFE, antibiotics, facial cleanliness, environmental improvement; S, surgery; TF, trachomatous inflammation-follicular; TT, trachomatous trichiasis.

## Historical perspective

In the 1990s, it was estimated that 590 million people lived in endemic areas, and there were 10.6 million people with TT^2^; these and subsequent estimates were based on extrapolation of data from country reports or were based on expert opinion. Population-based surveys led to the implementation of trachoma control programmes in some, but not all, districts mapped. In the calendar year 2011 it was reported that 96 000 people had received trichiasis surgical services and 44.8 million people received antibiotics for trachoma elimination purposes.^12^ The estimated number of trichiasis cases at the time (7.3 million) and the estimated number of people requiring antibiotics (325 million)^13^ suggested that the relatively piecemeal approach to mapping and intervention was not going to lead to elimination. The partnership between health ministries, WHO and supporting non-governmental organisations was too uncoordinated and patchy, causing significant gaps. Finally, it was recognised that the veracity of survey data was inadequate: some surveys were underpowered, sampling methodologies varied, there was a tendency to overgrade TF, and data cleaning and analysis approaches frequently had weaknesses.^14^

In 2011, under the sponsorship of the International Trachoma Initiative (ITI), a meeting of trachoma experts proposed that the ITI support three key initiatives: (1) the development of a standard trachoma survey tool, which ultimately led to the Global Trachoma Mapping Project (GTMP)^15 16^; (2) the development of a template for national planning for trachoma elimination, which became the Trachoma Action Plan (TAP)^17^; and (3) the production of a report detailing the current state of affairs on trachoma ("2020 INSight").^4^

### Trachoma mapping

In the 20 years prior to the GTMP, 1115 districts in 28 countries were mapped for trachoma, while in the approximately 3 years of the GTMP, from December 2012, 1546 districts in 29 countries were mapped.^18^ Key attributes of the GTMP’s approach to mapping included health ministry ownership, rigorous field-team training, sampling approaches tailored to local conditions while adhering to the WHO recommendations, grading of clinical signs by certified examiners, electronic data capture, and standardised analysis of findings by data managers independent of the programme itself.^14 16 19^ These things were made possible by the following:

- Enthusiasm by governments and partners to scale up trachoma mapping as quickly as possible.
- Broad agreement by scientists, partners, government and donors on how surveys would be undertaken.
- Careful planning, country by country, on all aspects of mapping.
- Complementary, coordinated financial support by UK aid (the UK government) and the United States Agency for International Development (USAID) for mapping.
- Support for national planning for trachoma elimination after GTMP data became available.

### Planning for trachoma elimination

The basic premise for planning for trachoma elimination was to make elimination the goal, requiring specific annual targets leading to the elimination target date. Essentially, annual targets for service delivery (trichiasis surgery and AFE activities) were set working back from the elimination target date. As of March 2018, TAPs have been completed in 25 of the 27 countries known to require interventions in sub-Saharan Africa. TAPs, which are intended to be living documents, updated as often as needed, are the trachoma component of the national NTD master plans. As national programmes near elimination, TAPs have provided the evidence for validation of elimination for national dossiers. Successful planning has been made possible by the following:

- Use of gold-standard GTMP data on trachoma prevalence at the district level in all suspected-endemic districts in the country.^20–22^
- Engagement of all partners with health ministries to develop a comprehensive, government-led approach to trachoma elimination.
- Experience from operational research and programmes, distilled into preferred practical manuals, to guide evidence-informed decision-making.
- Drafting of multiyear plans within a few days of the participatory TAP workshop.^17^

### Coordination and collaborative engagement for implementation

Elimination of trachoma as a public health problem is an attractive goal for governments, donors and partners, but it requires consensus that elimination is a distinct possibility, not just an aspiration. Clear messages regarding trachoma and elimination,^4^ combined with results from surveys and TAPs, made a compelling case for trachoma elimination, and large donors—UK aid from the UK government, USAID, The Queen Elizabeth Diamond Jubilee Trust and azithromycin (Zithromax) donated by Pfizer—have committed resources accordingly. Promised funding generally covers programmes through to elimination. Factors that led to the strong partnership include the following:

- Determined leadership by national trachoma programme managers for implementation and coordination of activities. Governments in endemic countries were keen to scale up as quickly as possible because funds were available.
- Estimates of unit costs for interventions (eg, for TT surgery and antibiotic mass drug administration) enabled partners to project the cost of elimination.
- Close collaboration between donors ensured that resources covered all components of the SAFE strategy.
- Members of the International Coalition for Trachoma Control (ICTC) agreed, as a group, that one member (Sightsavers) would serve as the ICTC grant manager for scale-up partnership programmes.
- TAPs outline, in clear detail, the annual targets for each programme component, providing transparent output indicators against which performance can be monitored.

## Future opportunities and challenges

Considerable progress has been achieved in the last 3 years, but some particular challenges remain.

### Reaching the end

The trachoma community greatly benefits from the experience of other large-scale disease elimination programmes, which demonstrate that the end game is often the hardest part of the whole endeavour. For example, finding and managing patients with TT is becoming progressively more difficult and expensive as prevalence falls. It is also anticipated that as active disease prevalence declines and mass drug administration comes to an end, there will be a misperception that the job is done. This will undoubtedly pose challenges; political and financial commitment may be more difficult to maintain.

### Creating stronger partnerships with the WASH sector

The organisations taking a lead on trachoma elimination are generally those whose mission and history align with provision of surgery and antibiotic interventions; they commonly lack indepth experience in water and sanitation (WASH) approaches. Establishing and strengthening partnerships with the WASH sector^23^ are essential to achieve elimination.

### Maintaining quality outcomes in TT surgery

Poor postoperative outcomes are a significant barrier to progress: they reduce the willingness of others to come for treatment.^24^ High-quality, high-volume surgery is needed to reach elimination targets, necessitating high-quality training and supervision of mid-level eye-care personnel. This may become more challenging as the prevalence of trichiasis falls: maintaining surgical skills is difficult without a steady case load, although mannequin-based training^25^ can help.

### Funding

Although efforts to eliminate trachoma benefit from global attention to NTDs, there remains a need to ensure a disease-specific focus on trachoma, because of its planned and defined end point. Due in part to generous trachoma-specific support, there is a misperception that trachoma work is fully funded; in fact, significant financial gaps remain for all components of the SAFE strategy and for monitoring and surveillance. As we near the elimination target, it will become increasingly difficult to ensure adequate levels of funding and government commitment. In this regard, continued coordination of existing donors, engagement of new donors, and increased advocacy at the national and subnational levels of endemic countries will be essential to achieving elimination.

## Footnotes

i The public health target for trachoma is elimination as a public health problem. For the sake of brevity, this will be referred to in this paper simply as ‘elimination’.
